# High Aerobic Intensity Training and Psychological States in Patients with Depression or Schizophrenia

**DOI:** 10.3389/fpsyt.2014.00148

**Published:** 2014-10-30

**Authors:** Jørn Heggelund, Kim Daniel Kleppe, Gunnar Morken, Einar Vedul-Kjelsås

**Affiliations:** ^1^Division of Psychiatry, Department of Østmarka, St. Olavs University Hospital, Trondheim, Norway; ^2^Department of Neuroscience, Faculty of Medicine, Norwegian University of Science and Technology, Trondheim, Norway; ^3^Hamar District Psychiatric Centre, Innlandet Hospital Trust, Ottestad, Norway; ^4^Division of Psychiatry, Department of Research and Development (AFFU), St. Olavs University Hospital, Trondheim, Norway

**Keywords:** exercise, intensity, affect, anxiety, transitory emotions

## Abstract

**Aim:** To explore changes in psychological states in response to a bout of high aerobic intensity training (HIT) in patients with depression or schizophrenia compared to healthy individuals.

**Methods:** After familiarization training of HIT, 20 patients with schizophrenia, 13 patients with depression, and 20 healthy individuals performed a no-training day followed by a training day. HIT was 4 × 4 min intervals at 85–95% of peak heart rate, intermitted by 3 min active rest periods at 70% of peak heart rate. Self-evaluation questionnaires of positive affect, negative affect, state anxiety, well-being, distress, and fatigue were completed before training, 15 min after, and 3 h after training. The two latter measures were also completed the no-training day.

**Results:** All three groups improved in positive affect and well-being 15 min after HIT (*p* < 0.01), but only patients with depression had maintained the effect after 3 h (*p* = 0.007, *p* = 0.012). The duration of the improved positive affect was longer in depression (*p* = 0.002) and schizophrenia (*p* = 0.025) than in healthy individuals (*F*_2.50_ = 5.83, *p* < 0.01). Patients with depression or schizophrenia had reduced distress and state anxiety 15 min after HIT and 3 h after HIT (*p* < 0.05). The improvement in distress 15 min after HIT was larger in patients with depression (*p* = 0.028) compared to healthy individuals (*F*_2.50_ = 5.05, *p* < 0.01). No changes were found during the no-training day (*p* > 0.05).

**Conclusion:** High aerobic intensity training used as an acute intervention improved positive affect and well-being and reduced distress and state anxiety in patients with depression and schizophrenia.

**ClinicalTrials.gov identifier:** NCT01310998.

## Introduction

Patients with severe mental illnesses suffer from long-term mental health problems that often persist in spite of extensive psychiatric treatments. Thus, acute affective improvements and relief of troubling symptoms may be beneficial, even though they would persist for a limited period of time. Improved self-reported affectivity such as mood enhancements, increased subjective well-being, decreased state anxiety, decreased negative affect, and decreased psychological stress may be brought about by aerobic endurance training in both healthy individuals and patients with severe mental illness (1–3). These affective improvements normally persist for 2–4 h following the cessation of exercise (4), but mood enhancements up to 12 h post-exercise have been reported (5).

To maximize the affective post-exercise improvements, it has been recommended that the exercise intensity is self-selected rather than prescribed (2, 6). Furthermore, increasing intensity is associated with reduced positivity of affect (7). These recommendations are somewhat in contrast to recommendations for increasing aerobic fitness in terms of peak oxygen uptake (VO_2peak_). High aerobic intensity training (HIT) performed as 4 × 4 min intervals at 85–95% of the maximal heart rate improves VO_2peak_ more than long slow distance training at 70% of the maximal heart rate (8–11). HIT also improves VO_2peak_ in patients with schizophrenia and could be used as an intervention to reduce the risk of cardiovascular disease in a high risk group, such as patients with severe mental illnesses (12–14). Despite a broad understanding of physiological mediators of improved VO_2peak_ after HIT, it is unknown whether HIT yields supplementary short-term psychological benefits. Long-term reductions in depressive score are plausible after regular HIT (14). Merely, a few studies have explored interval based exercise as an intervention to improve psychological states (7). Thus, it is reasonable to explore the influence of HIT on post-exercise psychological states.

The acute psychological benefits of aerobic exercise are widely explored in healthy individuals but to a lesser degree in patients with schizophrenia and depression. Patients with depressive disorders may score relatively high on negative affect, distress, fatigue, and state anxiety whereas low on positive affect and well-being, contrary to healthy individuals (15). Patients with depression may therefore have a larger window of improvement compared to healthy individuals. Decreased state-anxiety and negative affect along with improved positive well-being have been reported for patients with depression and anxiety (2, 16). Conversely, patients with schizophrenia may not be perceptive of psychological states and are known to have a lack of insight into their illness (17). Eight weeks of HIT did not improve chronic symptoms of schizophrenia (12). Acute improvements in state anxiety, psychological stress, and well-being have though been reported (3), and observations suggest that patients are less irritable, depressive, and psychotic as well as more socially interested and competent on the days they have exercised (18). Patients have also reported use of exercise as a coping strategy to deal with positive symptoms (19). HIT may therefore have important therapeutic benefits for patients with depression or schizophrenia. Thus, it is paramount to compare these groups to decide whether patients respond differently from healthy individuals and whether differences in responses exist between patient groups.

To explore the acute psychological benefits of HIT, we included patients with depression, schizophrenia, and healthy individuals to a controlled clinical trial. We hypothesized that a bout of HIT would improve psychological states 15 min and 3 h post-exercise, and patients would have greater acute benefits of HIT than healthy individuals.

## Materials and Methods

### Subjects

Fifty-three participants volunteered to take part in one of three study groups: (1) patients with schizophrenia or psychotic disorders, (2) patients with depression, or (3) healthy individuals. Patients were in- and out-patients at the University hospital psychiatric department. The schizophrenia/psychotic group was 20 patients with ICD-10 schizophrenia, schizotypal, or delusional disorders (ICD-10, F20–29) (20). The group of patients with depression comprised 1 with bipolar depression (F31.3), 11 with depressive episode (F32) or recurrent depression (F33), and 1 with dysthymia (F34.1). Twenty healthy controls with no history of psychiatric illness and with a training frequency less than three times per week were enrolled. Characteristics of the subjects are presented in Table 1.

**Table 1:** **Characteristics of the subjects**.

	Schizophrenia (*n* = 20)	Depression (*n* = 13)	Healthy (*n* = 20)
Men/women, *n*	13/7	8/5	10/10
Age (years), mean (SD)	37.7 (11.8)	41.0 (10.9)	40.8 (11.8)
Body weight (kg), mean (SD)	88.9 (17.3)	87.87 (26.6)	78.9 (14.2)
BMI (kg m^−2^), mean (SD)	24.79 (3.99)	24.95 (7.03)	22.42 (3.41)
Inpatient/outpatient, *n*	10/10	2/11	
ICD-10 diagnoses, *n*			
Schizophrenia	11		
Schizotypal disorder	1		
Delusional disorder	2		
Schizoaffective disorder	5		
Unspecified non-organic psychosis	1		
Bipolar depression		1	
Depressive disorder		11	
Dysthymia		1	

*Difference in inpatient/outpatient distribution between patient groups (*p* < 0.05)*.

### Instruments and procedures

During a period of two consecutive days, each participant completed self-report questionnaires’ about their psychological states. A bout of HIT was performed on the second day. Questionnaires’ were completed 10 min before (pre-) HIT, 15 min after (post-) HIT, and 3 h after HIT. The two latter measures were also completed at the same hour on the first day (no-training day). Participants were instructed to refrain from exercising 2 days before entering the data acquisition.

State anxiety was measured by the State-Trait Anxiety Inventory (STAI) form X-1 (21). A 12-item short form of the original 20-items was used (22). The STAI is a self-evaluation questionnaire and the instruction is to indicate how the subject feels at a particular moment in time. The scale ranges from 1 to 4. When evaluating the scores, six of the items are reversed so that higher scores indicate higher levels of anxiety. The test–retest correlations range from 0.16 to 0.54 in college students (21). A low level of reliability is expected since the scale reflects the influence of transient situational factors.

Positive affect and negative affect were measured by the Positive and Negative Affect Schedule (PANAS). The PANAS incorporates 10-items of positive affect and 10-items of negative affect. The scale ranges from 1 to 5 and higher score indicate higher perception of the factor. The subjects were asked to evaluate whether they experienced a particular emotion in the present moment. The positive affect and negative affect scores correlate negatively and positively, respectively, with the Beck Depression Inventory (BDI) (15, 23). Test–retest intraclass correlation is 0.79 and 0.93 for the positive and negative scales, respectively (24).

Positive well-being, psychological distress, and fatigue were measured by the Subjective Exercise Experiences Scale (SEES). SEES is a 12-item questionnaire that is validated to measure effects of exercise on psychological states (25). Each subscale contains the scores from four items and the scale ranges from 1 to 7. Higher scores indicate a higher perception of the particular factor.

Borg rating of perceived exertion (RPE) was applied to determine subjects’ perception of physical exertion during HIT and VO_2peak_ testing (26). The scale ranges from 6 to 20. Six indicate “no exertion at all” whereas 20 is “maximal exertion.”

### High aerobic intensity training

High aerobic intensity training was performed on a treadmill according to the procedures described elsewhere (8, 12). Four minutes intervals at a fixed workload were repeated 4 times, intermitted with 3 min active break periods between each interval. The intensity was 85–95% of peak heart rate (HR_peak_) during intervals and similar to warming-up work load (i.e., taxing about 70% of HR_peak_) during break periods. Subjects either walked or ran a minimum of 5% incline. Warm-up was 5 min. Heart rate was continuously measured using a Polar S610i heart rate monitor (Polar Electro, Finland). Heart rate and RPE was collected 3 min within each interval. All subjects performed a familiarization period of 3 HIT bouts within 2 weeks prior to the study. During familiarization training, the speed and incline were adjusted to attain the exact heart rate intensity (Table 2).

**Table 2:** **Mean (SD) work load of intervals during the high aerobic intensity training**.

	Schizophrenia (*n* = 20)	Depression (*n* = 13)	Healthy (*n* = 20)
Speed (km h^−1^)	7.4 (1.7)	7.2 (1.9)	8.5 (2.3)
Incline (%)	5.5 (1.3)	6.7 (3.4)	6.3 (1.0)
Watt	95 (29)	106 (45)	121 (29)

*No differences between groups*.

All subjects performed a test of HR_peak_ in order to calculate the HIT intensity (Table 3). The test was carried out as HIT interval training. The first interval was performed without an experience of leg stiffness. After a 3 min active rest, the second interval was carried out with >1 km h^−1^ incensements each minute until exhaustion (preferentially within 3–5 min). HR_peak_ was the highest recorded HR. A measure of blood lactate was taken 1 min after discontinuation using a Lactate Pro blood lactate test meter (Arkray, Inc, Japan). A peak test was accepted at a blood lactate level above 8 mmol L^−1^. Borgs RPE was collected at discontinuation of the test.

**Table 3:** **Mean (SD) values of the peak heart rate test**.

	Schizophrenia (*n* = 20)	Depression (*n* = 13)	Healthy (*n* = 20)
HR_peak_ (beats min^−1^)	177 (20)	186 (9)	186 (12)
Peak speed (km h^−1^)	9.4 (2.8)	9.5 (2.5)	11.4 (3.5)
Peak incline (%)	9.2 (3.8)	8.9 (3.8)	9.6 (3.8)
Watt	192 (75)	190 (86)	218 (79)
La^−^ (mmol l^−1^)	11.02 (3.60)	11.2 (2.2)	11.78 (3.16)
BORG RPE	19 (2)	19 (1)	19 (1)

*No differences between groups*.

*HR_peak_, peak heart rate; min, minute; RPE, rating of perceived exertion*.

### Statistical analyses

Within-group changes were analyzed using paired *t*-tests. One-way between-groups ANOVA was used to analyze baseline differences and differences in change between the three groups. Separate analysis were performed on the delta changes (pre- to 15 min post-HIT) and (pre- to 3 h post-HIT). LSD or Tamhane’s *post hoc* analyses were performed during significant main analysis. Cohens’s *d* effect sizes were calculated using the formula described in Nakagawa and Cuthill (27). The effect was considered small, medium, and large at 0.2, 0.5, and 0.8, respectively. Chi-square test was used to evaluate categorical data.

Statistical significance was reached at *p* < 0.05. Data are described as mean and SD, unless otherwise noted. IBM SPSS Statistics, version 20 (SPSS Inc.), was applied to analyze results.

The study was approved by the regional committees for medical and health research ethics, middle Norway and conducted according to the Helsinki declaration. Written informed consent was obtained from all the included patients after the procedures were fully explained.

## Results

Characteristics of the patients are presented in Table 1. There were no differences in age, bodyweight, or BMI between the groups (*p* > 0.05). There were no differences between the three groups in HR_peak_, speed, or incline during the peak treadmill test (*p* > 0.05; Table 3). All three groups performed the HIT at the prescribed intensity and duration. Work load of the intervals are presented in Table 2.

There were between-group differences at baseline in positive affect (*F*_2.50_ = 9.67, *p* < 0.01), negative affect (*F*_2.50_ = 17.21, *p* < 0.01), positive well-being (*F*_2.50_ = 19.84, *p* < 0.01), psychological distress (*F*_2.50_ = 12.64, *p* < 0.01), fatigue (*F*_2.50_ = 8.36, *p* < 0.01), and state anxiety (*F*_2.50_ = 21.74, *p* < 0.01). *Post hoc* analyses revealed that the positive affect (*p* = 0.046 and *p* < 0.001), negative affect (*p* = 0.005 and *p* < 0.001), psychological distress (*p* = 0.006 and *p* < 0.001), fatigue (*p* = 0.012 and *p* < 0.001), and state anxiety (*p* = 0.001 and *p* < 0.001) were different between health individuals and patients with schizophrenia and depression, respectively. Positive well-being was different between the healthy individuals and patients with depression (*p* < 0.001) but not between healthy individuals and patients with schizophrenia (*p* > 0.05). The patients with schizophrenia and patients with depression scored differently in positive affect (*p* = 0.013) and positive well-being (*p* < 0.001) but not in negative affect, psychological distress, fatigue, and state anxiety (*p* > 0.05) at baseline.

### Positive affect and positive well-being

**Figure 1:**
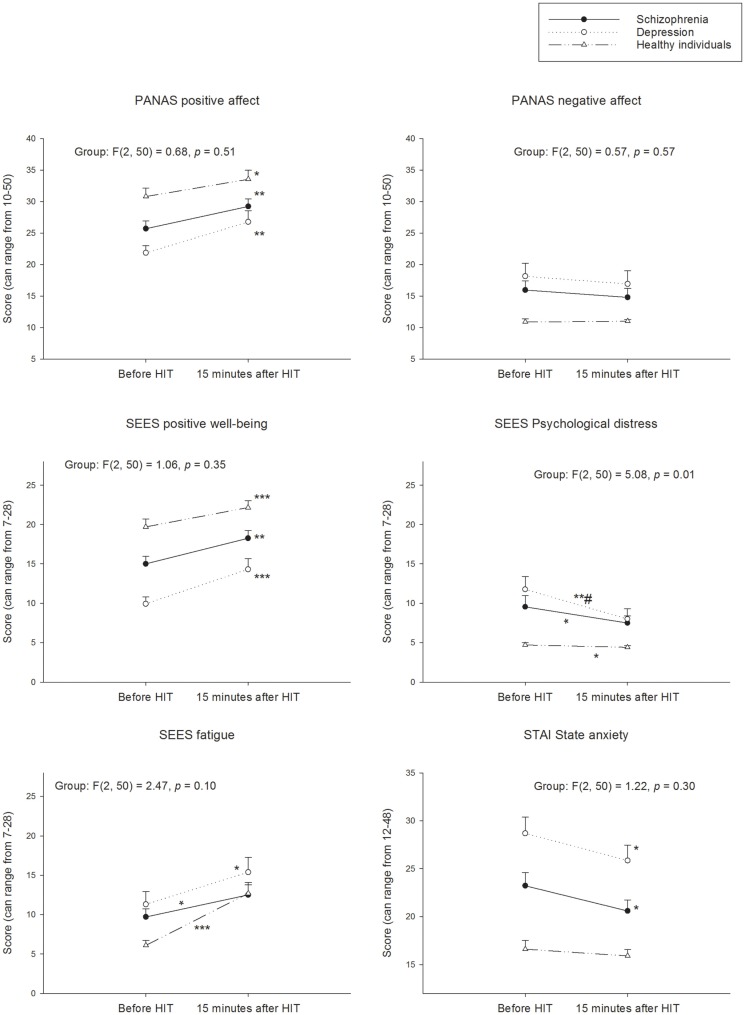
**Perception of psychological states before and 15 min after HIT**. PANAS, Positive and Negative Affect Schedule; SEES, Subjective Exercise Experiences Scale; STAI, State-Trait Anxiety Inventory Within groups; *p < 0.05; **p < 0.01; ***p < 0.001 different from before HIT. Between groups; different change from healthy ^#^*p* < 0.05.

**Figure 2:**
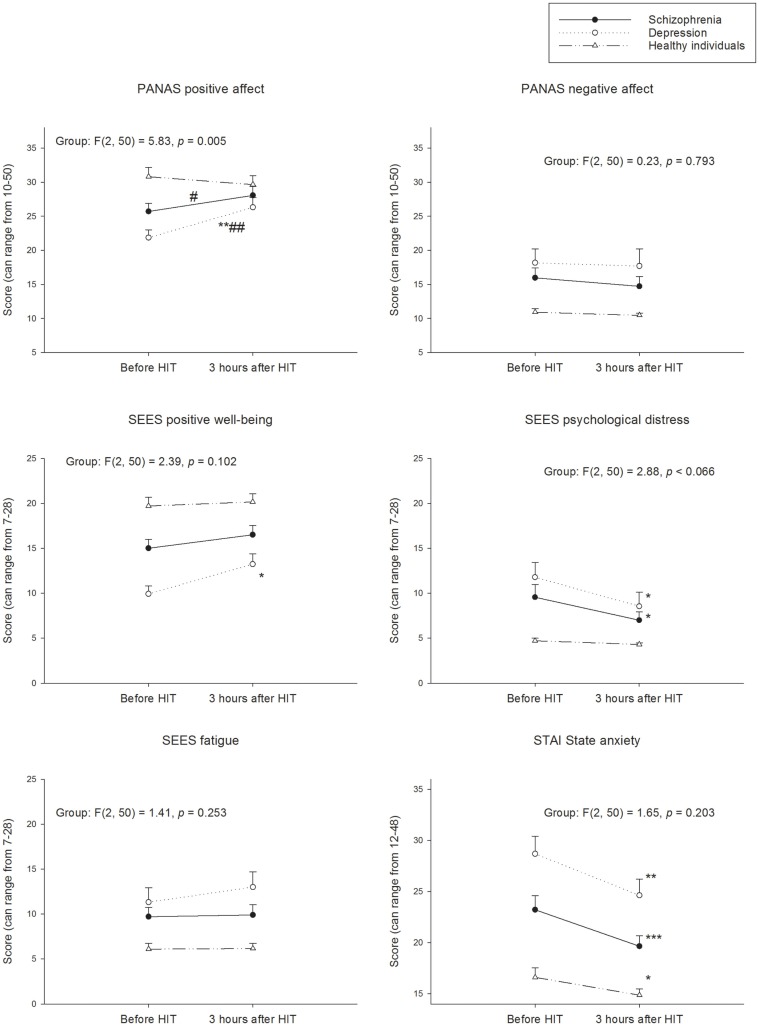
**Perception of psychological states before and after 3 h after HIT**. PANAS, Positive and Negative Affect Schedule; SEES, Subjective Exercise Experiences Scale; STAI, State-Trait Anxiety Inventory. Within groups; *p < 0.05; **p < 0.01; ***p < 0.001 different from before HIT. Between groups; different change from healthy ^#^*p* < 0.05; ^##^*p* < 0.01.

All three groups increased their experience of positive affect and well-being from pre- to 15 min post-HIT (Figure 1). The Cohen’s *d* effect size indicated a medium (0.66), large (0.91), and small (0.45) effect on the positive affect in the patients with schizophrenia, patients with depression, and healthy individuals, respectively. The corresponding Cohen’s *d* effect sizes for positive well-being was medium (0.74), large (1.05), and medium (0.58), respectively. After 3 h, the Cohen’s *d* effect sizes for positive affect and positive well-being were still large (0.96 and 0.90) in the patients with depression (Figure 2). The change in positive affect from pre-HIT to 3 h post-HIT was different between the groups (*F*_2.50_ = 5.83, *p* < 0.01), and the *post hoc* analyses revealed that the change in healthy individuals was different from patients with schizophrenia (*p* = 0.025) and patients with depression (*p* = 0.008).

### Psychological distress

The patients with schizophrenia and depression reduced their experience of psychological distress with a small (0.39) and medium (0.71) Cohen’s *d* effect size, respectively, from pre- to 15 min post-HIT (Figure 1). The Cohen’s *d* effect size for distress indicated a medium effect 3 h post-HIT (Figure 2) in the patients with schizophrenia (0.48) and depression (0.56). Cohen’s *d* effect size indicated a small effect (0.26) on distress in the healthy individuals, 15 min post-HIT. The three groups changed differently in distress from pre-HIT to 15 min post-HIT (*F*_2.50_ = 5.05, *p* < 0.01) and the *post hoc* analyses reviled a difference between the healthy individuals and the patients with depression (*p* = 0.028).

### State anxiety

The Cohen’s *d* effect sizes for state anxiety for patients with schizophrenia and patients with depression was medium (0.47 and 0.48) 15 min following the HIT (Figure 1). Cohen’s *d* indicated a medium (0.66) effect in patients with schizophrenia as well as in patients with depression (0.68). The healthy individuals had a higher level of state anxiety immediately before the HIT compared with the no-training day (*p* = 0.025; Cohen’s *d* = 0.59). This increase in state anxiety was unchanged from pre- to 15 min post-HIT (*p* = 0.408) but had returned to no-training day levels 3 h post-HIT as indicated by a reduction in state anxiety from pre-HIT values (*p* = 0.038; Cohen’s *d* = 0.50; Figure 2).

### Negative affect

The negative affect was unchanged following HIT in all groups (Figures 1 and 2). The negative affect was already reduced in the patients with depression before the HIT bout compared to the no-training day (*p* = 0.024; Cohen’s *d* = 0.59).

### No-training day

During the no-training day, the two measures were not different in either group (*p* > 0.05), which indicate a stable experience of all six psychological state variables.

Patients with schizophrenia (*p* = 0.038; Cohen’s *d* = 0.45), depression (*p* = 0.009; Cohen’s *d* = 0.81), and healthy individuals (*p* = 0.007; Cohen’s *d* = 0.48) experienced a lower level of fatigue before performing the HIT session compared to the no-training day.

## Discussion

The results from the present study support the hypothesis that supervised HIT improves the psychological state by increasing positive affect and well-being and reduce state anxiety and distress 15 min post-exercise in patients with schizophrenia and in patients with depression. The exact mechanism that led to the improved psychological state is not possible to elucidate from this study. Social, cognitive, and biological mechanisms of performing HIT in a supervised fashion in an Exercise Training Clinic contribute to the psychological response. However, when HIT is presented in this clinical wrapping, the high aerobic intensity falls within the range of intensities that improves psychological states beyond baseline levels already 15 min after cessation. The present study also finds that a prescribed intensity enhances the affective responses. Thus, it is possible to achieve these beneficial affective responses from prescribing an aerobic endurance training program that is developed for optimizing VO_2peak_. Exercising at a high aerobic intensity (i.e., 85–95% HR_peak_) is fatigable, as should be expected. All subjects rated the intervals in the range from somewhat hard – very hard (heavy), which is reasonably and at a fair distance from maximal exertion. The perception of fatigue, from the SEES questionnaire, is evident 15 min after cessation but is attenuated sometime within 3 h.

Patients with depression or schizophrenia sustained the reduction in distress and state anxiety more than 3 h after HIT but the improved positive affect and well-being did only persist in the patients with depression. The negative affect were unaltered in all groups at both of the post-HIT assessments. Together, these findings replicate previous results suggesting that the psychological benefits are sustained for some hours after the exercise is terminated (4, 5). The findings are tentative evidence that patients with depression might have particularly beneficial and sustainable responses in positive affect and well-being after HIT compared to the patients with schizophrenia and the healthy individuals. Patients with depression also have the lowest baseline scores in positive affect and well-being, which provides a large window of improvement. Positive affect and BDI score are in fact negatively correlated, which indicate that patients with enhancements in positive affect also would experience a reduction in BDI scores (23). Further, positive affect is more strongly related to depression than to anxiety (15). Thus, patients with high levels of depression might have important therapeutic benefits from HIT (16). Chamove (18) found a reduction in depression 2 h after exercise in patients with schizophrenia. Chamove (18) found that patients were “less tense” and “less irritable,” which probably reflect similar changes as the reduced distress and state anxiety in the present study. Chamove (18) applied a wider range of physical activities, illustrating that affective improvements likely appear through a multifactorial mechanism.

The between groups analyses confirm some beneficial responses in patients compared to healthy individuals. The duration of the improved positive affect was longer for those with depression or schizophrenia than in the healthy individuals. Further, the 15 min post-HIT effect on distress was larger in patients with depression compared to healthy individuals. These findings support the hypothesis that the benefits are largest in those with the most unbalanced affect and underscore the particular role of exercise in the treatment of patients with depression (28). It has been suggested that HIT does not improve chronic symptoms in patients with schizophrenia (12). Nevertheless, it now appears that patients are perceptive of certain affective improvements that exceed those of the healthy individuals. This might have important implications for daily functioning and quality of life, particularly at the days of exercise.

The psychological states were stable during the no-exercise day, which supports the influence of HIT. Further, the finding that some variables sustained elevated/reduced 3 h post-HIT also supports the hypothesis that they were influenced by HIT rather than being spontaneous changes. Studies also suggest that fitness level might influence the psychological response to exercise (7). VO_2peak_ measurements were not collected to explore exact differences between the three groups. However, the speed, incline, and watt load during the peak exercise test and the HIT intervals did not reveal any significant changes between the groups. We also assume that the familiarization training limited some of the affective responses of attending to an exercise regimen for the first time.

This study investigated supervised HIT at the hospitals Exercise Training Clinic. Thus, a combination of social, cognitive, and biological mechanisms likely has been responsible for the improvements (29). Performing supervised HIT exposes the patient to a range of psychosocial stimuli such as diversion from stressful stimuli, attention form the coach, improved self-image, feelings of control, social interaction, and social support (29). It is also suggested that exercise improves depression through a neurobiological mechanism. Regular exercise may exert a regulatory influence on the neurotransmitter system or/and affect the hypothalamic–pituitary–adrenal cortical (HPA) axis (29).

This study did not aim to compare HIT with other types of exercise interventions. Thus, it is not possible to advocate the use of HIT merely based on its effect on psychological states. Although the finding that a single HIT session is beneficial, it is appropriate to implement HIT on a regular basis in order to improve fitness and overall health. Better VO_2peak_ is associated with decreased depressive symptoms, quality of life, better capacity to cope with stress as well as reduced mortality from cardiovascular disease (30–33). It is evidential that HIT performed as 4 × 4 min intervals induces larger improvements in VO_2peak_ compared to other aerobic endurance training interventions (8–11). This large improvement caused by HIT contribute risk reduction of cardiovascular disease (12, 34) and likely influence depressive score (14). Thus, HIT should be implemented in clinical practice to improve VO_2peak_ in patients with severe mental illnesses.

## Conclusion

Supervised HIT performed at the Hospitals Exercise Training Clinic increased positive affect and well-being and reduced state anxiety and distress. It is therefore possible that patients could have twofold benefits from HIT. Training effectively to improve VO_2peak_ gave acute psychological benefits. Reductions in distress and state anxiety were sustained for more than 3 h after HIT and patients with depression also sustained the improved positive affect and well-being. The duration of the improved positive affect was longer for those with depressive and schizophrenia disorders than in the healthy individuals. The reduction in distress was also larger in patients with depression compared to healthy individuals with a low distress at baseline. It is therefore reasonable to conclude that patients with reduced affectivity had benefits from HIT that exceeded those in the healthy individuals.

## Author Contributions

Jørn Heggelund, Kim Daniel Kleppe, Gunnar Morken, and Einar Vedul-Kjelsås took part in the concept and design of the study. Jørn Heggelund conducted data analyses and prepared the manuscript. Kim Daniel Kleppe recruited participants and collected data, performed preliminary analyses and preparation of the manuscript. Gunnar Morken assisted data analyses, interpretation, and preparation of the manuscript. Einar Vedul-Kjelsås assisted with data collection, data analyses, and interpretation, and was involved in the preparation of the manuscript. All authors approved the final manuscript.

## Conflict of Interest Statement

The authors declare that the research was conducted in the absence of any commercial or financial relationships that could be construed as a potential conflict of interest.
